# Albumin stimulates renal tubular inflammation through an HSP70-TLR4 axis in mice with early diabetic nephropathy

**DOI:** 10.1242/dmm.019398

**Published:** 2015-10-01

**Authors:** Huei-Fen Jheng, Pei-Jane Tsai, Yi-Lun Chuang, Yi-Ting Shen, Ting-An Tai, Wen-Chung Chen, Chuan-Kai Chou, Li-Chun Ho, Ming-Jer Tang, Kuei-Tai A. Lai, Junne-Ming Sung, Yau-Sheng Tsai

**Affiliations:** 1 Institute of Basic Medical Sciences, National Cheng Kung University, Tainan 701, Taiwan; 2 Institute of Clinical Medicine, National Cheng Kung University, Tainan 701, Taiwan; 3 Department of Medical Laboratory Science and Biotechnology, National Cheng Kung University, Tainan 701, Taiwan; 4 Department of Physiology, National Cheng Kung University, Tainan 701, Taiwan; 5 Division of Nephrology, Department of Internal Medicine, National Cheng Kung University Hospital, Tainan 704, Taiwan; 6 Department of Pathology, National Cheng Kung University Hospital, Tainan 704, Taiwan; 7 National Laboratory Animal Center, National Applied Research Laboratories, Taipei 115, Taiwan; 8 Division of Nephrology, Department of Internal Medicine, E-DA Hospital/I-Shou University, Kaohsiung 824, Taiwan; 9 R&D Center, NovoTaiwan Biotech, Taipei 238, Taiwan; 10 Research Center of Clinical Medicine, National Cheng Kung University Hospital, Tainan 704, Taiwan, Republic of China

**Keywords:** Diabetic nephropathy, Toll-like receptor, Tubular injury, Albuminuria, Damage-associated molecular pattern (DAMP)

## Abstract

Increased urinary albumin excretion is not simply an aftermath of glomerular injury, but is also involved in the progression of diabetic nephropathy (DN). Whereas Toll-like receptors (TLRs) are incriminated in the renal inflammation of DN, whether and how albumin is involved in the TLR-related renal inflammatory response remains to be clarified. Here, we showed that both TLR2 and TLR4, one of their putative endogenous ligands [heat shock protein 70 (HSP70)] and nuclear factor-κB promoter activity were markedly elevated in the kidneys of diabetic mice. A deficiency of TLR4 but not of TLR2 alleviated albuminuria, tubulointerstitial fibrosis and inflammation induced by diabetes. The protection against renal injury in diabetic *Tlr4^−/−^* mice was associated with reduced tubular injuries and preserved cubilin levels, rather than amelioration of glomerular lesions. *In vitro* studies revealed that albumin, a stronger inducer than high glucose (HG), induced the release of HSP70 from proximal tubular cells. HSP70 blockade ameliorated albumin-induced inflammatory mediators. HSP70 triggered the production of inflammatory mediators in a TLR4-dependent manner. Moreover, HSP70 inhibition *in vivo* ameliorated diabetes-induced albuminuria, inflammatory response and tubular injury. Finally, we found that individuals with DN had higher levels of TLR4 and HSP70 in the dilated tubules than non-diabetic controls. Thus, activation of the HSP70-TLR4 axis, stimulated at least in part by albumin, in the tubular cell is a newly identified mechanism associated with induction of tubulointerstitial inflammation and aggravation of pre-existing microalbuminuria in the progression of DN.

## Abstract (precis)

**Summary:** Activation of the HSP70-TLR4 axis by albumin in the tubular cell induces tubular inflammation and injury.

## INTRODUCTION

The clinical and pathological signs of early diabetic nephropathy (DN) are renal hypertrophy, mesangial expansion and glomerular basement membrane (GBM) thickening, and the presence of albuminuria (Breyer et al., 2005; Gross et al., 2005; Brosius et al., 2009). Although the glomerulus has been the focus of investigations into DN, tubulointerstitial inflammation and tubular injury are also major features. Thus, pathological changes in the tubulointerstitium are closely correlated with the magnitude of renal dysfunction and albuminuria (Gilbert and Cooper, 1999). It has been shown that tubular functional and morphological changes precede the onset of microalbuminuria in early DN (Thomas et al., 2005). Thus, the role of tubulointerstitial injury in the progression of DN cannot be neglected.

Although the effect of hyperglycemia on tubular damage is documented, increased albumin leakage might cause tubulointerstitial injury and progression of renal diseases (Burton and Harris, 1996; Remuzzi et al., 1997). For example, excess albumin has been shown to induce tubular phenotypic changes, apoptosis and production of inflammatory mediators (Ohse et al., 2006; Zoja et al., 2003). However, the detailed mechanism by which albumin induces tubular injury is not yet clear. Within the tubulointerstitium, renal tubules abundantly express innate immune receptors, including Toll-like receptors (TLRs), suggesting that they are ready to sense environmental changes and transduce the inflammatory signals upon renal injury (Batsford et al., 2011). In addition to components from pathogens, cell-surface TLRs, such as TLR2 and TLR4, monitor tissue homeostasis by sensing endogenous ligands, which are known as damage-associated molecular patterns (DAMPs; Miyake, 2007; Rubartelli and Lotze, 2007).

TLR2 and TLR4 contribute to the pathogenesis of inflammation-associated renal injury and kidney disease (Brown et al., 2007; Cunningham et al., 2004; Leemans et al., 2005; Pulskens et al., 2010). The levels of TLR2 and TLR4 increase in the monocytes of type 2 diabetic patients and in the kidneys of diabetic rats (Dasu et al., 2010; Li et al., 2010). Although recent studies have demonstrated that a deficiency of TLR2 or TLR4 attenuates the inflammatory response and the development of DN (Devaraj et al., 2011; Kuwabara et al., 2012; Lin et al., 2012), what is not clear is which stimuli, in addition to hyperglycemia, predominantly activate the TLR-related inflammatory response. Whether TLR2 or TLR4 plays a more important role in diabetic renal injury remains unclear. In this study, we hypothesized that endogenous ligands released by the stimulation of albuminuria activate tubular cell inflammation via TLR signaling, which in turn accelerates the development and increases the severity of renal injury in DN.

**TRANSLATIONAL IMPACT**

**Clinical issue**

Diabetic nephropathy (DN), a complication of diabetes, is the most common cause of end-stage renal disease. Increased urinary albumin excretion, a hallmark of DN, has been suggested to be involved in the progression of DN. Although Toll-like receptors (TLRs) are well-known determinants of renal inflammation in DN, whether and how albumin is involved in the TLR-related renal inflammatory response remains to be clarified. The authors hypothesized that endogenous ligands released in response to albuminuria induce tubular cell inflammation via TLR signaling, which in turn accelerates the development and increases the severity of renal injury in DN. To test this hypothesis, the authors used as model organisms TLR2- and TLR4-deficient mice, in which they induced diabetes experimentally.

**Results**

The authors found that deficiency of TLR4 but not of TLR2 alleviates several diabetes-induced changes, including albuminuria, tubulointerstitial fibrosis, kidney inflammation and tubular apoptosis. The protection against renal injury in diabetic *Tlr4^–/–^* mice is associated with reduced tubular injury rather than amelioration of glomerular lesions. In the search for putative endogenous ligands of TLRs, the authors found that heat shock protein 70 (HSP70) is markedly elevated in the damaged tubules of diabetic mice. Cell culture studies revealed that albumin can stimulate the release of HSP70 and is a stronger inducer of HSP70 than high glucose. Blockade of HSP70 attenuates albumin-induced expression of inflammatory mediators. Moreover, HSP70 induces the production of inflammatory mediators in a TLR4-dependent manner. To examine the clinical significance of these responses, the authors found that both TLR4 and HSP70 are dramatically upregulated in damaged tubules of kidneys from individuals with DN.

**Implication and future directions**

This study highlights the HSP70-TLR4 axis as a key mediator of tubular inflammation and emphasizes the potential contribution of albuminuria to tubular injury in DN. Thus, this work exemplifies how clinical observations can be dissected mechanistically via basic investigations in murine and cell models. Evaluating whether this mechanism also exists in other types of renal disease requires further studies. Nevertheless, the inhibition of tubular inflammation with agents that target the albumin-HSP70-TLR4 axis might represent a new therapeutic strategy to halt progression of DN in humans.

## RESULTS

### Activation of nuclear factor-κB and inflammatory response in the diabetic kidney

**Fig. 1.**
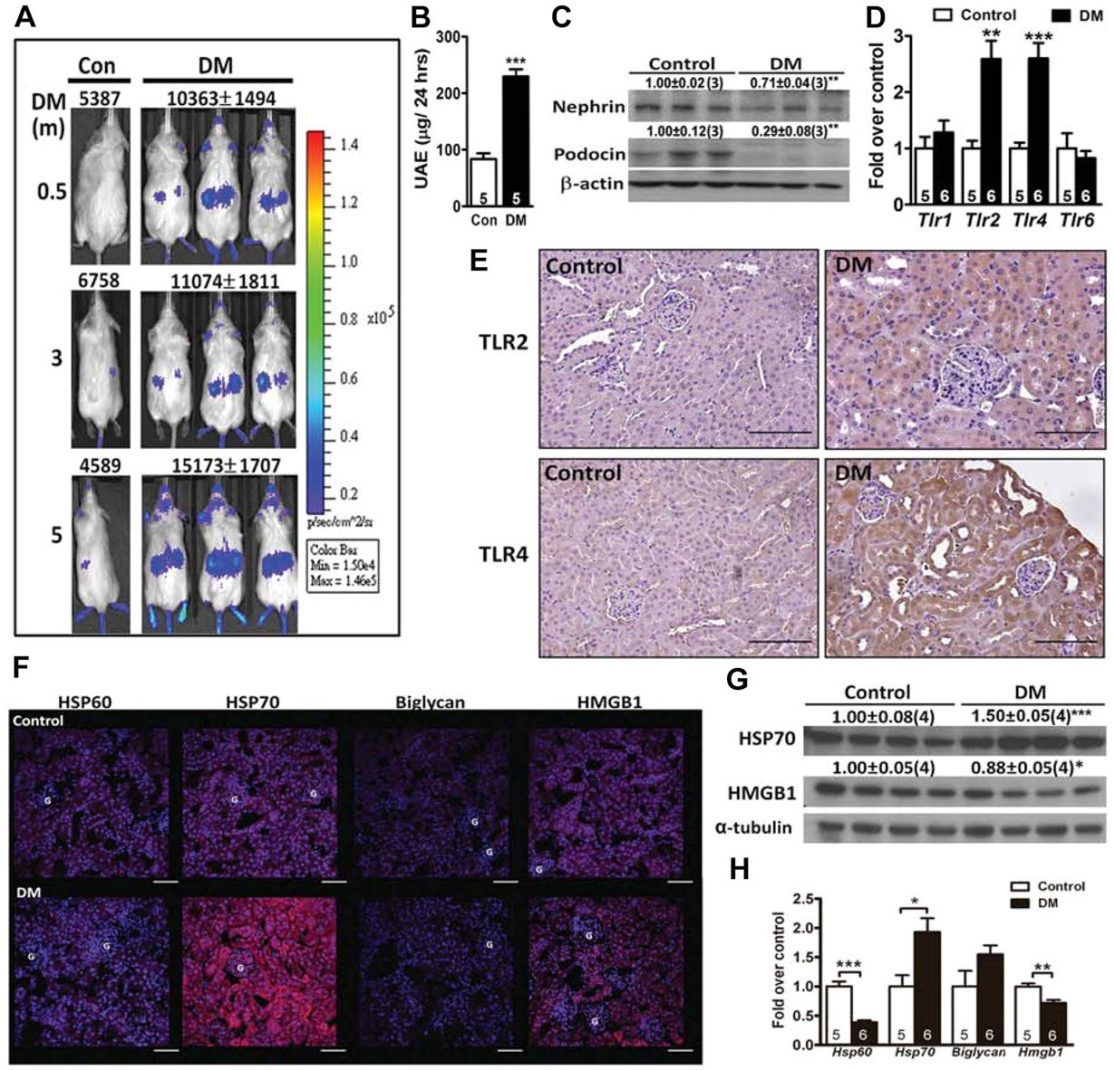
**Expression and localization of inflammatory mediators in the diabetic kidney.** (A) Imaging and photon counting in diabetic and control male transgenic (NF-κB-RE-luciferase) mice that were diabetic for 0.5, 3 or 5 months. The color overlays on the images represent the photons per second emitted, and the quantified photon signals are shown. Daily urinary albumin excretion (UAE; B) and immunoblot analyses (C) on nephrin and podocin from the kidney of 1-month-diabetic C57BL/6 mice. The relative intensities of the bands are indicated by densitometric quantification compared with control mice, with the number of mice in parentheses. (D) Expression of TLRs in the kidney of 1-month-diabetic relative to control mice. Immunohistochemical staining for TLR2 and TLR4 (E) and immunofluorescence staining for HSP60, HSP70, biglycan and HMGB1 (red) (F) in the kidney of 1-month-diabetic and control mice. The DAPI nuclear counterstain appears blue*.* Scale bars: 50 µm. G, glomerulus. (G) Immunoblot analyses on HSP70 and HMGB1 from the kidney of 1-month-diabetic C57BL/6 mice. (H) Expression of DAMPs in the kidney of 1-month-diabetic relative to control mice. *P<0.05, **P<0.01 and ***P<0.001.

To assess the inflammatory status directly *in vivo*, we induced diabetes by intraperitoneal injection of streptozotocin (STZ) into nuclear factor-κB (NF-κB)-luciferase reporter mice. Two weeks after STZ induction, an increase of luminescence was detected in the abdominal region of diabetic mice, and the signal lasted for 5 months after the induction of diabetes (Fig. 1A). Expression of luciferase was increased in the kidney, but not in the liver and lung (Fig. S1). Consistently, increased expression of cytokines, chemokines and macrophage markers, associated with elevated urinary albumin excretion (UAE) and downregulated nephrin and podocin levels, was found in the kidney of 1-month-diabetic C57BL/6 mice (Fig. 1B,C and Fig. S1). Furthermore, induction of diabetes in wild-type (WT) mice for 3 months resulted in early features of DN, including elevated UAE, tubulointerstitial fibrosis, mesangial matrix expansion and GBM thickening (Fig. S2).

### Upregulation of TLRs and HSP70 in the diabetic kidney

The expression of *Tlr2* and *Tlr4* was 2.5-fold higher in the diabetic kidney than in non-diabetic controls, whereas they were not different in the liver and lung (Fig. 1D and Fig. S3). The diabetic kidney exhibited increased expression of TLR2 and TLR4 predominantly in the tubules, without apparently increased expression in the glomeruli (Fig. 1E). The increased TLR4 expression was more prominent in the dilated proximal tubules, with thinning or loss of the brush border in the kidney of diabetic mice. In addition, diabetes significantly increased the level of HSP70, whereas the levels of HSP60 and biglycan were not affected (Fig. 1F). Although high-mobility group box 1 (HMGB1) has been shown to be involved in DN (Lin et al., 2013, 2012), its upregulation was relatively mild. Furthermore, the increased HSP70 and HMGB1 protein levels were predominantly located in the tubules of the diabetic kidney. Consistently, immunoblotting analysis also confirmed a significant upregulation of HSP70, but no change in HMGB1 (Fig. 1G). Gene expression of *Hsp70*, but not other genes, was significantly upregulated in the diabetic kidney (Fig. 1H). Thus, a diabetic milieu predominantly increased HSP70 and marginally upregulated HMGB1, and these molecules might serve as endogenous ligands for TLRs.

### Attenuation of albuminuria in *Tlr4^−/−^* diabetic mice

To address the functional significance of TLR2 and TLR4 in the pathogenesis of DN directly, *Tlr2^−/−^* and *Tlr4^−/−^* mice were used. Susceptibility to STZ induction was not influenced by the lack of either TLR2 or TLR4, evidenced by the similar blood glucose levels (Tables S1 and S2). UAE, urinary albumin-to-creatinine ratio and tubulointerstitial fibrosis were significantly attenuated in *Tlr4^−/−^* diabetic mice (Fig. S4), but they did not differ between *Tlr2^−/−^* and WT diabetic mice (Fig. S5). However, we did not find significant differences in mesangial matrix expansion, GBM thickening, and nephrin and podocin levels between *Tlr2^−/−^*, *Tlr4^−/−^* and WT diabetic mice (Figs S4 and S5). Thus, a deficiency in TLR4 but not TLR2 attenuates diabetes-induced albuminuria and tubulointerstitial fibrosis, which is not associated with significant improvements in the structural and molecular changes in the glomerulus.

### Attenuation of inflammatory response in *Tlr4^−/−^* diabetic mice

The kidney of 1-month-diabetic *Tlr4^−/−^* mice showed significantly lower expression of chemokines, macrophage marker, and profibrotic genes (Fig. S4). Macrophage infiltration was significantly compromised in *Tlr4^−/−^* diabetic kidney (Fig. S4). However, these parameters were not different between *Tlr2^−/−^* and WT diabetic kidney (Fig. S5). These results suggest that the improved renal function in *Tlr4^−/−^* diabetic mice is associated with decreases of macrophage infiltration and expression of key genes for profibrotic and pro-inflammatory mediators.

### Reduction of tubular injury in *Tlr4^−/−^* diabetic mice

**Fig. 2.**
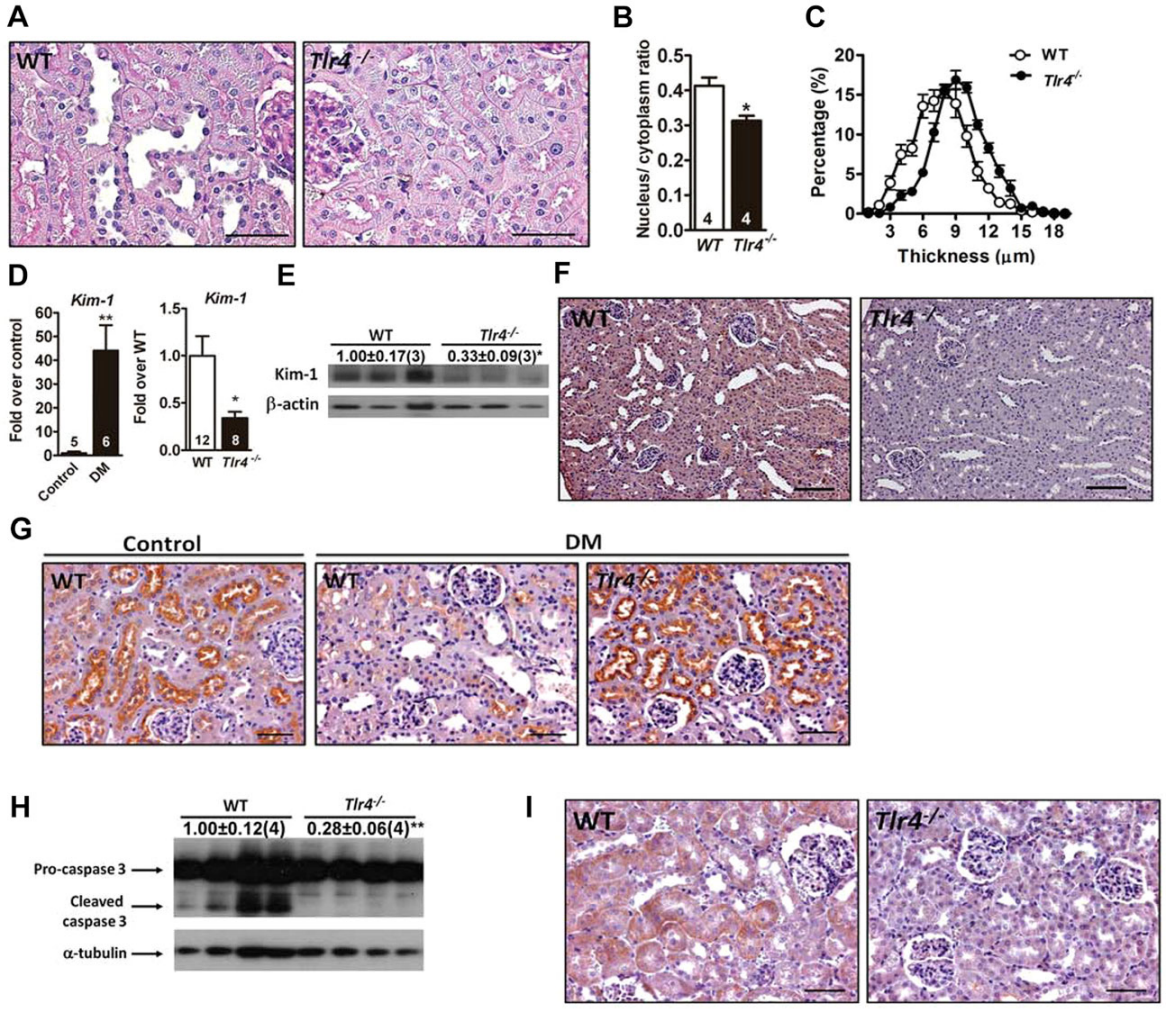
**Renal tubular injury and apoptosis in *Tlr4^−/−^* diabetic mice.** Representative tubular morphology (A), quantification of the tubular nucleus-to-cytoplasm ratio (B) and distribution of tubular epithelial thickness (C) in *Tlr4^−/−^* and WT 1-month-diabetic mice. Scale bars: 50 µm. (D) Expression of *Kim-1* in WT control mice and WT and *Tlr4^−/−^* 1-month-diabetic mice. Numbers inside the bars indicate the number of mice in each group. Immunoblot analyses (E) and immunohistochemical staining (F) of Kim-1 in the kidney of *Tlr4^−/−^* and WT 1-month-diabetic mice. Scale bars: 100 µm. (G) Immunohistochemical staining of cubilin in the kidney of WT control mice and WT and *Tlr4^−/−^* 1-month-diabetic mice. Scale bars: 50 µm. Immunoblot analyses (H) and immunohistochemical staining (I) of caspase 3 in the kidney of 1-month-diabetic mice. The relative intensities of the bands are indicated by densitometric quantification with WT, with the number of mice in parentheses. Scale bars: 50 µm. *P<0.05 and **P<0.01.

The kidney of 1-month-diabetic *Tlr4^−/−^* mice showed less tubular pathological change, evidenced by reductions of tubular dilatation, brush border loss and flattened tubular epithelium (Fig. 2A). Quantification revealed a decreased nucleus-to-cytoplasm ratio and an increased epithelial thickness in *Tlr4^−/−^* diabetic kidney (Fig. 2B,C). Furthermore, we found that diabetes dramatically induced kidney injury molecule 1 (Kim-1), a marker for proximal tubular injury, and downregulated cubilin, a receptor for albumin uptake (Fig. 2D-G). TLR4 deficiency significantly blunted Kim-1 and preserved apical cubilin levels in the diabetic kidney. Caspase-3 activation in the kidney, particularly within the tubules, was attenuated in diabetic *Tlr4^−/−^* mice (Fig. 2H,I). In contrast, diabetic *Tlr2^−/−^* and WT mice showed similar degrees of tubular damage and apoptosis (Fig. S6). These results suggest that TLR4 plays a dominant role in renal tubular injury in the diabetic condition.

### Albumin induces NF-κB activation and HSP70 release in LLC-PK1 cells

**Fig. 3.**
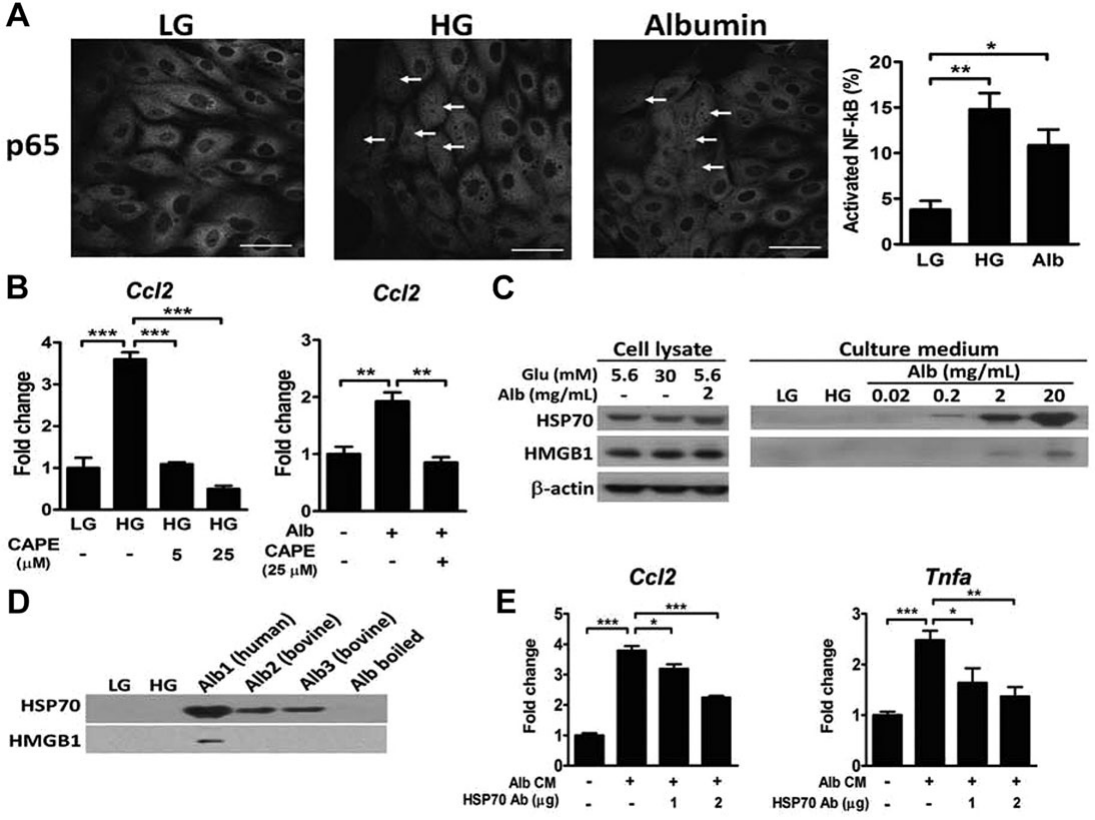
**Effects of HG and albumin on NF-κB activation and DAMP release in LLC-PK1 cells.** (A) Immunofluorescence staining of NF-κB in LLC-PK1 cells after 24 h incubation in medium containing 5.6 mM glucose (LG), 30 mM glucose (HG) or 5.6 mM glucose with 2 mg/ml albumin. The percentage of NF-κB translocation into the nucleus is presented. *n*=3 in each group. Scale bars: 10 μm. (B) Expression of *Ccl2* in LLC-PK1 cells treated with LG, HG or albumin with or without caffeic acid phenethyl ester (CAPE) for 24 h. *n*=3 in each group. (C) Immunoblot analyses of HSP70 and HMGB1 from the cell lysate and culture medium of LLC-PK1 cells treated with LG, HG and different concentrations of albumin for 24 h. (D) Immunoblot analyses of HSP70 and HMGB1 from the culture medium of LLC-PK1 cells treated with various sources of albumin (2 mg/ml). Alb1, fatty acid free human albumin; Alb2, fatty acid free bovine albumin; Alb3, essential fatty acid free bovine albumin; Alb boiled, Alb3 boiled for 10 min. (E) Expression of *Ccl2* and *Tnfα* in LLC-PK1 cells treated with the conditioned medium (CM) with or without depletion of HSP70 for 8 h. *P<0.05, **P<0.01 and ***P<0.001 by one-way ANOVA followed by Dunnett's test.

Treatment of porcine proximal tubular LLC-PK1 cells with HG and albumin significantly increased the expression of *Ccl2*, *Tnfα* and *Mip2* (data not shown) and translocation of NF-κB into the nucleus (Fig. 3A). Co-treatment with an NF-κB inhibitor, caffeic acid phenethyl ester, attenuated the increased *Ccl2* expression (Fig. 3B). We next examined DAMP production and release. HSP70 and HMGB1 levels were not different in the lysates of LLC-PK1 cells treated with low glucose (LG), HG and albumin (Fig. 3C). In contrast to the proposed effect of HG on tubular cells (Lin et al., 2012; Mudaliar et al., 2013), only albumin efficiently induced HSP70 release into the medium, accompanied by a slight release of HMGB1. The effect of albumin on HSP70 secretion was independent of species differences and contamination with fatty acid, but was abolished by boiling (Fig. 3D); however, only human albumin was able to stimulate HMGB1 release from LLC-PK1 cells. Finally, depletion of HSP70 by antibodies significantly attenuated albumin-induced expression of *Ccl2* and *Tnfα* in LLC-PK1 cells (Fig. 3E).

### HSP70 mediates production of albumin-induced inflammatory mediators in mouse proximal tubular cells

**Fig. 4.**
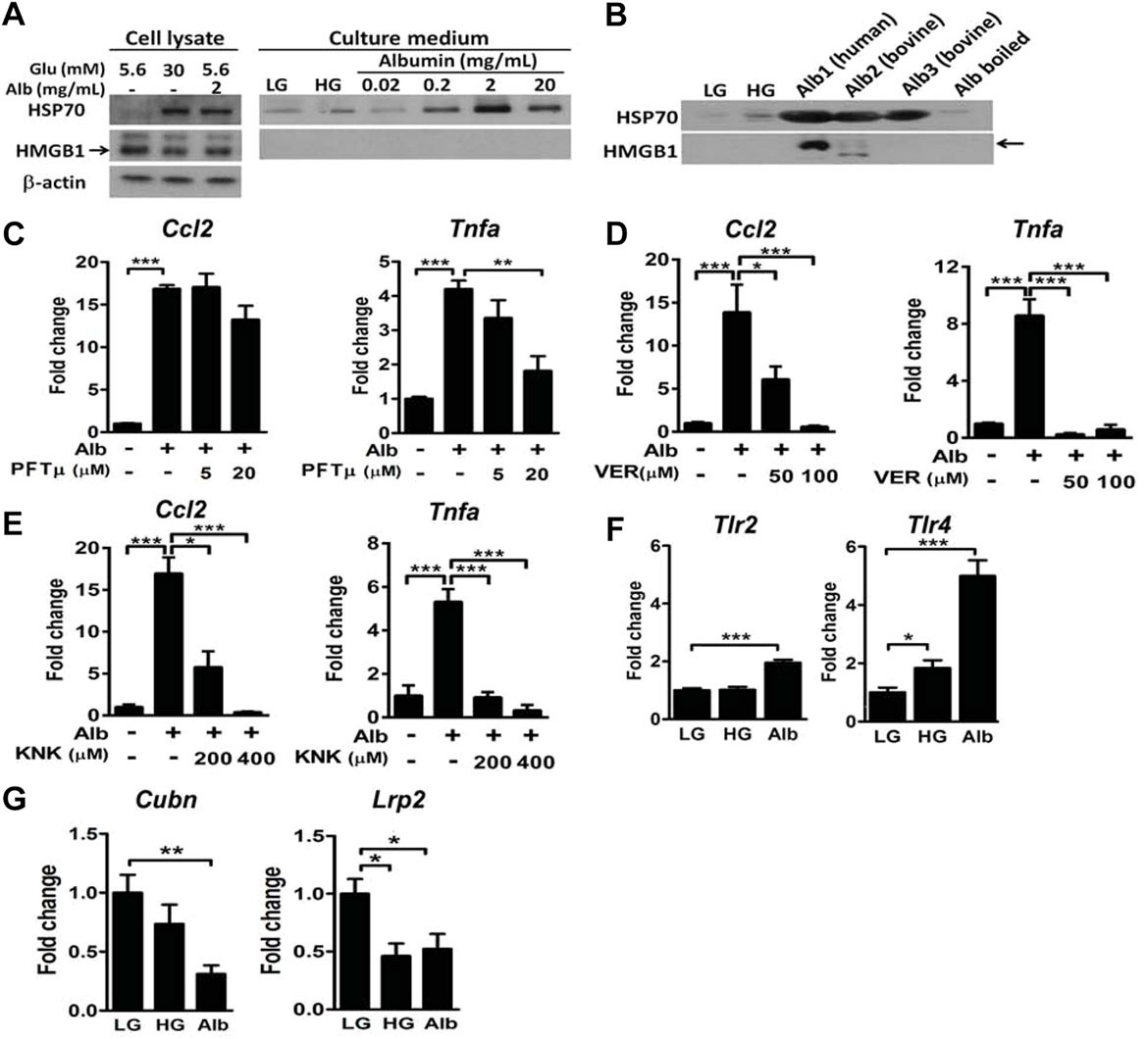
**Effects of HG and albumin on DAMP release and gene expression in mPTCs.** (A) Immunoblot analyses of HSP70 and HMGB1 from the cell lysate and culture medium of mPTCs treated with LG, HG and different concentrations of albumin for 24 h. (B) Immunoblot analyses of HSP70 and HMGB1 from the culture medium of mPTCs treated with various sources of albumin (2 mg/ml) as described for Fig. 3D. Expression of *Ccl2* and *Tnfα* in WT mPTCs pretreated with pifithrin-μ (C), VER-155008 (D) and KNK437 (E) for 30 min before albumin stimulation. *n*=3-4 in each group. Expression of *Tlr2* and *Tlr4* (F) and *Cubn* and *Lrp2* (G) in WT mPTCs treated with LG, HG and albumin (0.2 mg/ml) for 24 h. *P<0.05, **P<0.01 and ***P<0.001 by one-way ANOVA followed by Dunnett's test.

In a primary culture of mouse proximal tubular cells (mPTCs), HG and albumin treatments significantly induced the production of HSP70, but not HMGB1 (Fig. 4A). Although HG caused a marginal induction of HSP70 release, albumin at a concentration as low as 0.2 mg/ml efficiently induced HSP70 release from mPTCs. In the same conditions, HMGB1 was not detectable regardless of the stimulation. Moreover, both human and bovine albumins stimulated HSP70 release from mPTCs, but only human albumin was able to stimulate HMGB1 release (Fig. 4B). HSP70 blockade by various inhibitors, including pifithrin-μ (PFTμ), VER-155008 (VER) and KNK437 (KNK), attenuated albumin-induced expression of *Ccl2* and *Tnfα* in mPTCs (Fig. 4C-E). We also examined the effects of HG or albumin on changes of TLRs and endocytic receptors. TLR2 was upregulated only by albumin, whereas TLR4 was dramatically upregulated by albumin and modestly by HG in mPTCs (Fig. 4F). Additionally, cubilin was downregulated only by albumin, whereas megalin (encoded by the gene of low-density lipoprotein receptor-related protein) was downregulated by both HG and albumin (Fig. 4G). These results suggest that albumin plays a major role in induction of HSP70 release, upregulation of TLRs and downregulation of endocytic receptors in the tubule.

### TLR4 mediates HSP70-induced production of inflammatory mediators

**Fig. 5.**
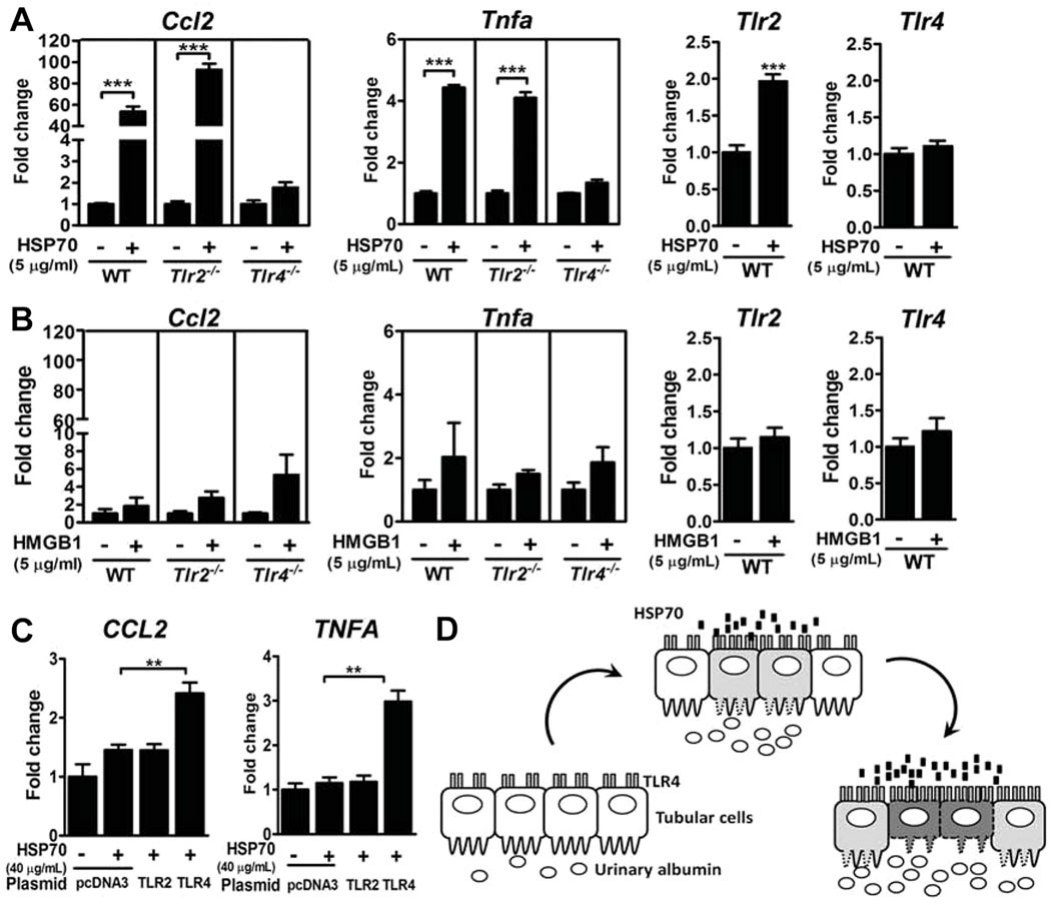
**TLRs in DAMP-induced inflammatory response.** Expression of *Ccl2*, *Tnfα*, *Tlr2* and *Tlr4* in mPTCs from *Tlr2^−/−^*, *Tlr4^−/−^* and WT mice treated with 5 µg/ml human HSP70 (A) and 5 µg/ml human HMGB1 (B) for 8 h. ***P<0.001 by Student's *t*-test. (C) Expression of *CCL2* and *TNFA* in TLR2- and TLR4/CD14/MD2-overexpressing HEK293T cells treated with 40 µg/ml human HSP70 for 8 h. **P<0.01 by one-way ANOVA followed by Dunnett's test. (D) The proposed model of the albumin-HSP70-TLR4 axis in renal tubular inflammation.

We next addressed which TLR mediated the DAMP-induced inflammatory response in the tubular cell. Treatment with HSP70 at 5 μg/ml increased expression of *Ccl2* and *Tnfα* in the mPTCs from WT and *Tlr2^−/−^* but not *Tlr4^−/−^* mice (Fig. 5A). HSP70 treatment also increased TLR2 expression, but had no effect on TLR4 expression. Although HMGB1 has been suggested to activate TLRs (Mudaliar et al., 2013), HMGB1 at this concentration was not able to induce significant increases of *Ccl2*, *Tnfα*, *Tlr2* and *Tlr4* (Fig. 5B). To confirm whether TLRs are sufficient to mediate the stimulation from HSP70, we reconstituted TLR2 or TLR4 in human embryonic kidney (HEK) 293T cells. HEK293T cells overexpressing TLR2 or TLR4 successfully responded to their specific agonists, Pam3CSK4 and lipopolysaccharide (data not shown). However, treatment with HSP70 increased expression of *CCL2* and *TNFα* in TLR4-expressing cells, but not in TLR2-expressing cells (Fig. 5C). These results suggest that HSP70 triggers the production of inflammatory mediators in a TLR4-dependent manner. The proposed model of the albumin-HSP70-TLR4 axis in the renal tubular inflammation is summarized in Fig. 5D.

### HSP70 blockade *in vivo* ameliorates diabetes-induced albuminuria, inflammatory response and tubular injury

**Fig. 6.**
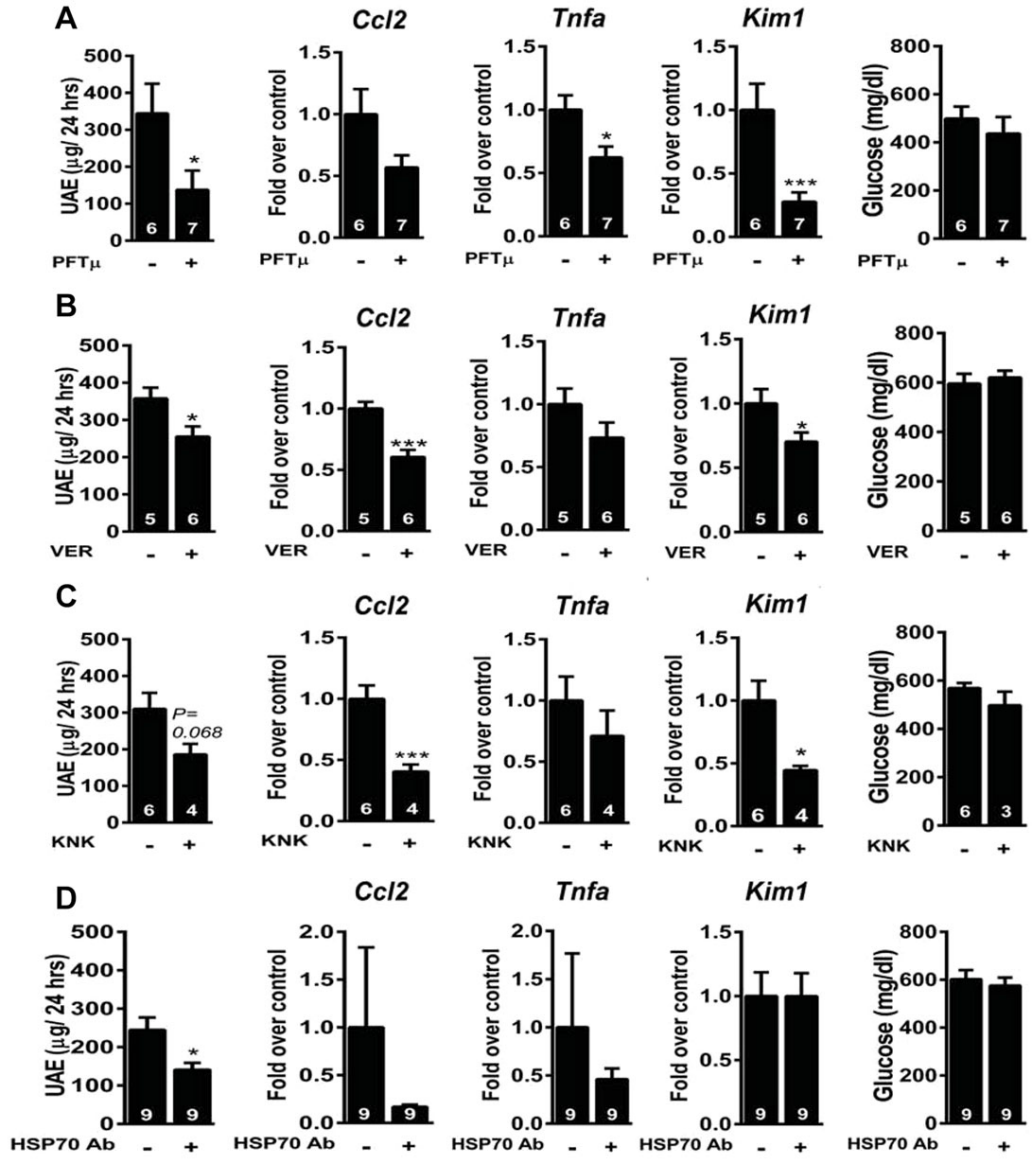
**HSP70 blockade in DN mice.** UAE, expression of *Ccl2*, *Tnfa* and *Kim1* and concentrations of glucose in 2-week-diabetic mice that received pifithrin-μ (5 mg/kg; A), VER-155008 (16 mg/kg; B), KNK437 (25 mg/kg; C) or anti-HSP70 or isotype-matched IgM antibodies (D) intraperitoneally for 1 week. *P<0.05 and ***P<0.001 by Student's *t*-test.

We next applied two cell-permeable inhibitors (PFTμ and VER), one HSP70 transcriptional inhibitor (KNK) and one neutralizing anti-HSP70 antibody in DN mice. Treatment with PFTμ and VER attenuated UAE and expression of *Ccl2*, *Tnfα* and *Kim1*, without a change in glucose concentration (Fig. 6A,B). Transcriptional inhibition of HSP70 by KNK also attenuated UAE and expression of *Ccl2*, *Tnfα* and *Kim1* (Fig. 6C). Functional antagonism of extracellular HSP70 by a neutralizing anti-HSP70 antibody decreased UAE and tended to decrease expression of *Ccl2* and *Tnfα* (Fig. 6D). Therefore, HSP70 inhibition *in vivo* is protective against diabetes-induced albuminuria, inflammatory response and tubular injury.

### Expression of TLRs and DAMPs in renal biopsies from patients with DN

**Fig. 7.**
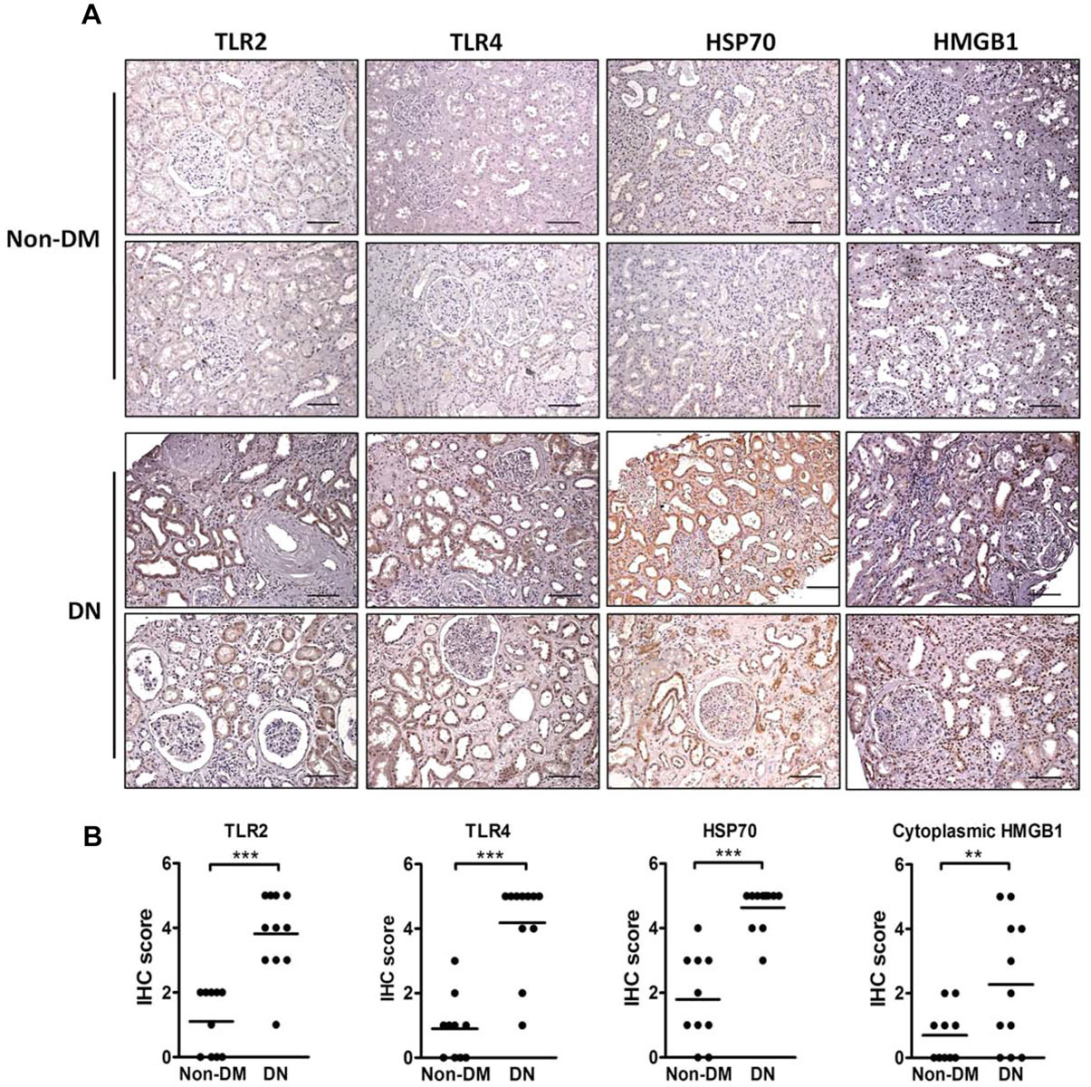
**Renal expression of TLR2, TLR4, HSP70 and HMGB1 in human biopsies.** Representative photomicrographs (A) and scoring (B) of TLR2, TLR4, HSP70 and HMGB1 immunohistochemical staining in the renal tissue from DN patients (*n*=11) and non-diabetic controls (Non-DM, *n*=10). Scale bars: 200 µm. **P<0.01 and ***P<0.001.

Finally, we assessed the expression of TLR2, TLR4, HSP70 and HMGB1 in the kidney tissues from patients with DN and non-diabetic controls. Although TLR2 and TLR4 were modestly expressed in the tubules of non-diabetic controls, they were both robustly increased in the dilated tubules of DN biopsies (Fig. 7 and Table S3). Consistently, HSP70 was significantly upregulated in the tubules, especially in dilated tubules, of DN biopsies. Nuclear HMGB1 staining was observed in both DN and control biopsies, whereas cytoplasmic HMGB1 staining was significantly increased in the tubules of DN biopsies.

## DISCUSSION

Accumulating evidence indicates that inflammation plays a significant role in the development and progression of DN (Navarro-Gonzalez and Mora-Fernandez, 2008). The increased luminescence signal in our transgenic mice unequivocally demonstrated that NF-κB is activated at the initial stage, and this activation persists through the development of DN. Furthermore, our results showed that HSP70, TLR2 and TLR4 were upregulated in the kidney of diabetic mice and human patients with DN. The *in vitro* study showed that albumin, which is a stronger inducer than HG, induced upregulation of both *Tlr2* and *Tlr4* and a significant release of HSP70 into the culture medium. TLR4, rather than TLR2, mediated the HSP70-induced upregulation of inflammatory cytokines in the tubular cell. Thus, we propose a mechanism that, in the early stage of diabetes, a leakage of albumin into the renal tubules upregulates TLR2 and TLR4 and induces HSP70 release and TLR4 activation, leading to a more severe injury and a stronger inflammatory response in the tubule and interstitium (summarized in Fig. 5D).

There has been much focus on the change in glomerular permeability as the primary factor in the kidney for the manifestation of albuminuria (Molitch et al., 2004). Instead, other studies have emphasized the importance of diminished albumin uptake by the tubules in governing albuminuria in diabetes (Russo et al., 2009; Tojo et al., 2001). It is possible that the albuminuria that develops in diabetes might be a consequence of impairments of both the glomerular filtration barrier and tubular reabsorption. As we did not find significant improvements of glomerular structure and podocyte functional molecules in *Tlr4^−/−^* diabetic mice, we speculated that amelioration of albuminuria in *Tlr4^−/−^* diabetic mice might be a consequence of an improvement in tubular reabsorption. Given that albumin molecules are taken up by the megalin-cubilin complex receptor located on the apical surface of proximal tubules (Gekle, 2005), the tubular injury would impair albumin reabsorption (Christensen et al., 2012). In addition, mice exhibiting downregulation of megalin or cubilin show proteinuria or albuminuria (Leheste et al., 1999; Liu et al., 2011). The function and quantity of megalin and cubilin are reduced in early DN (Kaseda et al., 2011; Tojo et al., 2001). Our results showed that diabetes markedly upregulated Kim-1 and downregulated cubilin. A deficiency of TLR4 but not of TLR2 blunted the increased Kim-1 level and tubular injury score and preserved the apical cubilin level. These results suggest that TLR4 deficiency reduces the proximal tubule injury, which, in turn, contributes to albumin recovery.

Recently, Russo et al. (2007) have shown that even in normal conditions nephrotic levels of albumin (∼1 mg/ml) are filtered through the GBM. Therefore, the apical side of proximal tubules should be exposed to a high concentration of albumin even in healthy conditions without overt inflammatory responses. The process of albumin-induced tubular inflammation involves the initial receptor-mediated endocytic uptake of albumin (Birn and Christensen, 2006). Endocytosed albumin is transported across the tubular cell (transcytosis) or transferred to lysosomes for degradation and release into the cytosol. Excessive binding of extracellular albumin to megalin might initiate intracellular signaling events, including NF-κB activation (Wang et al., 1999). How great the capacity of normal proximal tubule is to handle an increasing albumin load before tubular injury or stress could develop is still controversial. One study showed that lysosomal enzyme activity and membrane permeabilization were preserved in HK-2 cells loaded with 2 mg/ml urinary proteins (Liu et al., 2015). Currently, we do not have good explanations for the lack of inflammation if proximal tubules face a high apical albumin load. It is reasonable to speculate that healthy tubules might preserve lysosomal function or signaling desensitization machineries to prevent further stress response and signaling. Nevertheless, proximal tubules are exposed on their basolateral side to blood via the peritubular capillaries, where much albumin (∼30 mg/ml) is present. No overt inflammatory responses in this situation can be explained by the predominantly apical location of the receptors for albumin (Birn and Christensen, 2006; Tang et al., 2003).

Several studies have documented the involvement of TLR4 in development of DN. For example, Kuwabara et al. (2012) showed a predominant activation of S100A8/TLR4 signaling in the glomeruli of diabetic mice. However , the principal site for TLR4 expression in diabetes is the renal tubules, which might react in response to different stimuli. The role of TLR4 in tubular inflammation was shown by genetic and pharmacological approaches (Lin et al., 2012, 2013). Both studies proposed that HG induces a pro-inflammatory effect using a cell model. Although our results also showed that HG induced NF-κB activation and increased *Tlr4* expression in proximal tubular cells, no apparent releases of HSP70 and HMGB1 were detected. Importantly, while similar blood glucose levels were reached in the STZ-only and STZ+uninephrectomy groups of the study by Lin et al. (2012), the induction of TLR4 and inflammatory response were exacerbated in their STZ+uninephrectomy group. These results suggest that factors other than HG, possibly contributed by enhancing hyperfiltration, might induce the release of DAMPs and more severe inflammatory response. Given that uninephrectomy has been shown to hasten the development of DN and albuminuria, we thus speculated that albumin is a trigger for tubular inflammation. Our results consistently showed that albumin treatment induced NF-κB nuclear translocation, production and release of DAMP, and production of inflammatory mediators in the proximal tubular cells. The putative role of urinary albumin in inducing tubulointerstitial changes is also supported by others (Eddy, 1989; Tang et al., 2003). Although the concentration of albumin in the proximal convoluted tubule in diabetes has been debated (Oken and Flamenbaum, 1971; Tojo and Endou, 1992), we found that treatment of mPTCs with pathophysiologically relevant concentrations of albumin for 24 h induced these changes. These results suggest that albuminuria is not simply an aftermath of glomerular injury, but can directly cause tubular inflammation and renal injury.

In the search for putative endogenous ligands for TLR activation, HMGB1 has been suggested for activation of TLRs in DN (Mudaliar et al., 2013). Our results also showed that HMGB1 was slightly increased in the kidney of diabetic mice, and cytoplasmic HMGB1 staining was significantly upregulated in DN biopsies. We next tested the causative relationship in a cell model and found that HMGB1 was minimally detected in the medium of LLC-PK1 and mPTC cells treated with albumin and could not be detectable in those treated with HG. Moreover, HMGB1 at 5 μg/ml could not efficiently upregulate inflammatory mediators in mPTCs. Thus, the role of HMGB1 in progression of DN remains speculative. In contrast, we found that HSP70 was upregulated in mPTCs and released by LLC-PK1 cells and mPTCs treated with albumin. HSP70 blockade attenuated albumin-induced production of inflammatory mediators. HSP70 triggered the production of inflammatory mediators in a TLR4-dependent manner. Finally, HSP70 was significantly upregulated in the kidney of diabetic mice and patients with DN. Although the upregulation of tubular HSP70 in DN patients is controversial (Calabrese et al., 2007; Nakhjavani et al., 2010; Lin et al., 2012), selection bias, study population and individual diversity might be responsible for the discrepancies. Nevertheless, our results establish a causative relationship among albumin, HSP70 and TLR4 in tubular inflammation during progression of DN.

Intracellular HSP70 is generally considered to be cytoprotective and anti-inflammatory, whereas extracellular HSP70 functions as DAMP and is pro-inflammatory. Therefore, it is important to discriminate the role of intracellular and extracellular HSP70 in DN. Two cell-permeable inhibitors, VER and PFTμ, are proposed to target different sites in HSP70 (Schlecht et al., 2013; Zhang et al., 2013). VER, which binds to the nucleotide binding site of HSP70, acts as an ATP-competitive inhibitor. PFTμ, in spite of its inhibition of p53, interferes with the substrate binding domain of HSP70 and disrupts its association with client proteins. These two compounds are thought to inhibit intracellular HSP70. KNK437, a specific inhibitor of HSF-1, inhibits the transcription of HSP70 (Cai et al., 2010), thus decreasing production of both intracellular and extracellular HSP70. In contrast, a neutralizing anti-HSP70 antibody acts only on extracellular HSP70 (Cai et al., 2010). As the efficiency of delivery of anti-HSP70 neutralizing antibody to the apical side of tubular cells could be lower than other small molecule compounds, the effect of extracellular HSP70 antagonism is relatively weaker. Nevertheless, our results showed that blockade of both intracellular and extracellular HSP70 ameliorated diabetes-induced albuminuria, inflammatory response and, perhaps, tubular injury. Recently, it has been shown that HSF-1-deficient mice with an absent stress response are protected against ischemic renal injury, confirming the contribution of intracellular HSP70 to ischemic renal injury (Sreedharan et al., 2014).

The role of TLR2 in the renal injury of DN has recently been investigated. Our finding of modestly increased TLR2 expression in the kidney of diabetic mice and human DN biopsies is consistent with the previous study (Li et al., 2010). Devaraj et al. (2011) demonstrated that TLR2 knockout attenuates the renal inflammatory state and incipient DN. Although we found some restoration of the increased inflammatory cytokines in *Tlr2^−/−^* diabetic mice, most of these changes were modest. Our findings suggest that the effect of TLR2 deficiency on protection from renal injury in DN is relatively modest. The discrepancy between these two studies might stem from the insulin supplementation and STZ dosage/frequency. The lower number of animals in our TLR2 study might not be sufficient to detect a significant difference. Nevertheless, because the phenotypic characterization of *Tlr2^−/−^* and *Tlr4^−/−^* mice was carried out simultaneously in our study, it is reasonable to compare the magnitude of renal injury and inflammatory status between them. Our results showed that a deficiency of TLR4 had more beneficial effects than that of TLR2, suggesting that activation of the TLR4 signaling pathway plays a more important role in diabetic renal injury than that of TLR2.

In conclusion, our findings identify TLR4 as a critical mediator of inflammatory responses that lead to renal injury and dysfunction in DN. Blocking of TLR4-mediated inflammatory responses attenuated the development of albuminuria, partly through decreases in factors associated with fibrosis, the inflammatory response and tubular injury in the kidney. *In vitro* studies of tubular cells established a causative effect of albumin on inducing the release of HSP70 and the expression of inflammatory mediators via a TLR4-dependent pathway. Thus, leaked albumin results in a cycle of chronic tubular injury, inflammation and dysfunction through activation of the HSP70-TLR4 axis. Our study highlights the HSP70-TLR4 axis as a key mediator of tubular inflammation and emphasizes the potential contribution of albuminuria to tubular injury in DN.

## MATERIALS AND METHODS

### Animals

*Tlr2^−/−^* and *Tlr4^−/−^* mice were kindly provided by Dr S. Akira (Osaka University) and maintained on a C57BL/6 genetic background. Diabetes was induced in 8-week-old male mice by intraperitoneal administration of STZ (Sigma-Aldrich) at 65 mg/kg body weight for five consecutive days. Mice with a fasting blood glucose >300 mg/dl were considered diabetic. For HSP70 blockade *in vivo*, pifithrin-μ (5 mg/kg; Calbiochem), VER-155008 (16 mg/kg; Sigma) and KNK437 (25 mg/kg; Sigma) were administered intraperitoneally to 2-week diabetic mice every other day for 7 days. For antibody blocking, 2-week diabetic mice were injected intraperitoneally with either anti-HSP70 or isotype-matched IgM antibodies (25 μg/kg; Novus) every other day for 7 days. Mice were fed *ad libitum* with regular chow (Purina Laboratory Rodent Diet 5001; PMI Nutrition International). Animals were housed in a specific-pathogen-free barrier facility and were handled in accordance with procedures approved by the Institutional Animal Care and Use Committee of National Cheng Kung University.

### *In vivo* imaging system

The generation of NF-κB-luciferase reporter mice was carried out by injection of a linearized plasmid with four NF-κB-responsive elements upstream of firefly luciferase cDNA (4x-κB-*luc*) into FVB fertilized oocytes, and maintained on an FVB genetic background. Mice were anesthetized and imaged with an ultrasensitive camera (IVIS Spectrum; Xenogen). Signal intensity was measured as the sum of all detected photon counts per second within the region of interest after subtracting the background luminescence and is presented as the number of photons per second per square centimetre per steradian.

### Renal function

For the assessment of renal function, mice were housed in metabolic cages (Solo Mouse Metabolic Cage; Tecniplast) for 24 h, and water intake and urine output were recorded. Urinary albumin concentration was measured by immunoassay (Bethyl Laboratories), and urine creatinine was determined by the Jaffe enzymatic method.

### RNA analysis

Tissues were stored in RNAlater (Ambion), and total RNA was extracted with REzol (Protech Technology). Samples of mRNA were analyzed by SYBR Green-based real-time quantitative RT-PCR, with *β-actin* as the reference gene in each reaction.

### Immunostaining and immunoblotting analyses

For the immunohistochemical staining, paraffin-embedded sections (5 µm thick) were incubated with primary antibodies against mouse TLR2 (Abcam), TLR4 and cubilin (Santa Cruz), Kim-1 (R&D) and cleaved caspase 3 (Cell Signaling); or human TLR2 and TLR4 (Santa Cruz), HSP70 (Enzo Life Science) and HMGB1 (Abcam). Slides were developed using 3,3′-diaminobenzidine substrate-chromogen solution (Dako). For the immunofluorescence staining, frozen sections (12 µm thick) were incubated with antibodies against HSP60 and HSP70 (Enzo Life Science), and biglycan and HMGB1 (Abcam) followed by secondary antibodies conjugated with Alexa Fluor dyes (Invitrogen). For the immunobloting, proteins were probed with antibodies against Kim-1, podocin, HSP70 (Santa Cruz), HMGB1 (Abcam), nephrin (PROGEN), caspase 3 (Cell Signaling), β-actin and α-tubulin (Sigma-Aldrich).

### Image quantification

The ratio of nucleus to cytoplasm in tubules was calculated as the area of hematoxylin (nucleus staining) divided by the area of background staining (cytoplasmic staining) in five to ten fields of renal cortex per mouse. The thickness of tubular epithelium was measured on a line across the apical/basolateral tubular interface, and the average was based on >500 tubules examined per mouse. The quantification was performed using image analysis software (AxioVision; Zeiss). NF-κB activation was examined with p65 immunofluorescence staining using a confocal microscope (C1-Si; Nikon). The percentage of p65-positive cells was calculated as the number of cells showing nuclear red staining divided by the total number of cells from three images per group. The expression of TLR2, TLR4, HSP70 and HMGB1 in the cortex of renal biopsies was graded on the following scale from 0 to 5: 0 (negative), 1 (1-20% positive), 2 (21-40% positive), 3 (41-60% positive), 4 (61-80% positive) and 5 (>80% positive; Lin et al., 2012).

### *In vitro* study

The mPTCs were isolated according to a method described previously (Chen et al., 2014) and maintained in a 1:1 Dulbecco's modified Eagle's medium (DMEM):Ham's F12 medium containing 10% fetal bovine serum and 17.5 mM glucose. LLC-PK1 and HEK293T cells were maintained in low-glucose DMEM supplemented with 10% fetal bovine serum.

For the treatments of glucose and albumin, LLC-PK1 cells and mPTCs were preconditioned with low-glucose DMEM containing 0.1% fetal bovine serum for 24 h and then treated with low glucose (5.6 mM), high glucose (30 mM) or albumin (0.02-20 mg/ml in 5.6 mM glucose) for 24 h. Various sources of albumin, including human (Alb1, Sigma #A3782) and bovine albumin (Alb2, Sigma #A8806 and Alb3, #A6003), were used. Unless otherwise defined herein, bovine albumin (Sigma #A6003) was used. The endotoxin levels in all albumin preparations were less than 0.03 EU/ml, determined by an endotoxin detection kit (Pyrotell).

For HSP70 depletion, the culture medium from LLC-PK1 cells was incubated with anti-HSP70 antibody (Santa Cruz) or control IgG overnight at 4°C, followed by incubation with Protein G beads (GenScript) for 2 h. The supernatant was used for the subsequent experiments. For HSP70 inhibition, mPTCs were pretreated with pifithrin-μ, VER-155008 and KNK437 for 30 min followed by albumin treatment for 24 h.

For DAMP stimulation, mPTCs from TLR2- or TLR4-deficent mice were stimulated with 5 µg/ml human HSP70 (Enzo Life Science) or HMGB1 (Sigma) for 8 h.

For TLR overexpression, confluent HEK293T cells were transfected with TLR2 (90 ng) or TLR4/CD14/MD2 plasmids (30 ng of each) for 24 h and then changed to medium with 40 µg/ml human HSP70 or HMGB1 for 8 h.

### Human renal tissues

Renal biopsies diagnosed as DN without evidence of other pathological changes were obtained from 11 diabetic patients. Ten normal kidney tissues obtained from non-diabetic patients who received nephrectomy for renal tumor were used as the controls. Their clinical data are shown in Table S3. Renal biopsies from patients who had a history of diabetes and a final histological diagnosis of DN without other types of kidney disease, confirmed by microscopic, immunofluorescent and electron microscopic examinations, were used as the DN group. The control renal tissues were obtained by searching the tumor registry at the Human Biobank of National Cheng Kung University Hospital for patients who underwent nephrectomy for a renal tumor. These ten subjects had no medical history of diabetes and all had fasting blood glucose lower than 126 mg/dl. The control tissues from normal renal tissues adjacent to the tumor without other renal diseases except for the solitary renal tumor were confirmed by microscopic examination. All subjects provided written informed consent for this study, which was approved by the Institutional Review Board of National Cheng Kung University Hospital.

### Data analysis

Values are reported as means±s.e.m. Statistical analyses were conducted by Student's *t*-test or one-way ANOVA followed by Dunnett's multiple comparison test. Differences were considered to be statistically significant at *P*<0.05.
